# Coexistence of Biopsychosocial Factors With Lumbopelvic Pain in Indian Women: A Systematic Review

**DOI:** 10.7759/cureus.36937

**Published:** 2023-03-30

**Authors:** Priyanka Sushil, Jasmine Kaur Chawla, Raju K Parasher

**Affiliations:** 1 Amity Institute of Health Allied Sciences, Amity University, Noida, IND; 2 Amar Jyoti Institute of Physiotherapy, University of Delhi, Delhi, IND

**Keywords:** indian women, musculoskeletal, psychological, biological, lumbopelvic pain

## Abstract

In general, women appear to report lumbopelvic pain (LPP) more frequently. In addition to the biomechanical risks, this systematic review aimed to identify the add-on biopsychosocial implications of LPP among women in the Indian community. PubMed, ScienceDirect, Web of Science, PEDro, and Google Scholar were searched twice from inception to a final systematic literature search in December 2022. All studies addressing Indian women with LPP were selected. Studies on non-musculoskeletal LPP were excluded. Qualities of non-experimental and experimental research articles were assessed through the *Critical Appraisal Skills Programme (CASP)* checklist and *Cochrane risk of bias criteria *for Effective Practice and Organization of Care reviewsrespectively. Data synthesis was narrative as the selected studies differed substantially. Habitual squatting, kneeling, and continuous sitting were identified as ergonomic risks to LPP. Menopause, cesarean, and multiple deliveries influence the onset of LPP among women. There is a severe deficit in data about the musculoskeletal implications of LPP. There are insufficient data present to summarize the biopsychosocial risks of LPP. Even the exact anatomical sites of LPP were not described in most articles. Due to the severe scarcity of data, there is an alarming need to explore the musculoskeletal as well as psychosocial consequences of LPP in Indian women. Among rural women, LPP was common in those working as laborers; which are physically robust jobs with respect to strength and anthropometrics of women. Domestic chores in India involve a lot of manual work; placing unequal loads on the lumbar spine, eventually resulting in LPP. Therefore ergonomic strategies for women should be designed to meet the needs and demands of their respective occupations as well as domestic chores.

## Introduction and background

Owing to musculoskeletal lumbopelvic pain (LPP), women face significant rehabilitative, social, and financial inferences throughout the world [1,2]. The likelihood of developing chronicity of LPP also appears to be high among women [1,3,4]. Hence, LPP in women is a crucial musculoskeletal concern for healthcare professionals. Even the World Health Organization (WHO) has stressed the importance of spine care in everyday activities [5]. However, rural India is still unaware of non-pharmacological treatments for musculoskeletal LPP [6]. The plight of women is worse in such Indian communities, as they are barely allowed to access essential health screenings [7]. When instead, women are in dire need of musculoskeletal screenings [8,9]. Globally, women are frequent visitors for the rehabilitation of LPP [4]. However, in some Indian communities, misogyny is an obstacle for women in seeking healthcare [10]. It won’t be surprising if Indian women are coping with unattended LPP disabilities.

Disability secondary to LPP impedes workplace performance and consequently inflicts substantial costs to health and quality of life. To minimize these costs, several industries came up with various innovative strategies in order to prevent LPP in workplaces. Ergonomic and general spine care interventions are quite popular in the prevention of occupation-based injuries to the musculoskeletal system in the west [11]. These strategies could help the rising number of Indian women in the agriculture field; however, ergonomic-based equipment designed for ease of manual workforce may not match with anthropometrics and physical strength of women [12]. Further, physical ergonomic-based interventions alone are not effective in preventing LPP [13] compared to participatory ergonomic strategies [14]. Hence, LPP takes a toll on the work and social life of afflicted women [15]. To develop an efficient ergonomic-based program for the prevention of LPP; one must be updated on the risks contributing to LPP [11].

The reduced work productivity on account of LPP also expedites the psychological well-being of women already bearing LPP. As per the definition by the International Association for the Study of Pain, pain is an ‘unpleasant sensory and emotional experience’ that is associated with actual impairment [15]. Thus, LPP is partially an emotional experience, which somehow is associated with the psychological well-being of an individual. Unfortunately, women have high odds of psychological distress in response to various social, familial, work, and health issues that indirectly influence LPP. Women with LPP have self-reported the worsening of symptoms when burdened with a domestic workload.

Having a thorough understating of the possible risks of LPP in women will add insights to women-specific rehabilitation. Hence, the purpose of this review was to consolidate studies addressing Indian women experiencing LPP to gather information in terms of parameters like the clinical presentation and risk factors of lumbopelvic pain.

## Review

Methodology

This systematic review was in adherence to Preferred Reporting Items for Systematic Reviews and Meta-Analyses (PRISMA) guidelines *(Review protocol registered at PROSPERO: CRD42021227044 on January 23, 2020).*

Ethical Considerations

This review is in accordance with research ethical standards and doesn’t involve animal and human subjects.

Search Strategy

PubMed, ScienceDirect, Web of Science, PEDro, and Google Scholar were searched from inception up to August 2022 followed by an updated search on December 2, 2022. Boolean operators “AND” and “OR” were combined with keywords ‘lumbago’, ‘low back pain’, ‘lumbar pain’, ‘pelvic pain’, ‘chronic pelvic pain’, ‘pelvic girdle pain’, ‘lumbopelvic pain’, ‘India’, and women’ (Table 1). A literature search was not restricted to any particular language; however, all studies were found in the English language, and translations were not required. Searched citations retrieved from the databases were merged and duplicates were removed. Full texts of articles were scrutinized by two authors for final inclusion in the review.

**Table 1:** Search strategy

Search strategy
Free-text search words (as well as MESH terms) Lower back pain, pelvic pain, Lumbago, Backache, India, women with required AND or OR Boolean operators in the searches.
MEDLINE and Cochrane Library databases (PubMed) search using MeSH terms #1 (lower back pain[MeSH Terms]) AND "India"[MeSH Terms]) AND women #2 ("back pain"[MeSH Major Topic]) AND "India"[MeSH Terms]) AND "women"[MeSH Terms] #3 (pelvic girdle pain[MeSH Major Topic]) AND Indian women[MeSH Terms] #4 (sacroiliac joint dysfunction[MeSH Major Topic]) AND "women"[MeSH Terms]) AND "India"[MeSH Terms] #5 (lumbago[MeSH Major Topic]) AND "women"[MeSH Terms]) AND "India"[MeSH Terms] #6 (lumbar pain[MeSH Major Topic]) AND "women"[MeSH Terms]) AND "India"[MeSH Terms] #7 ("pelvic pain"[MeSH Major Topic]) AND "women"[MeSH Terms]) AND "India"[MeSH Terms] #8 (sacroiliac pain[MeSH Major Topic]) AND "women"[MeSH Terms]) AND "India"[MeSH Terms]
Science direct chronic low back pain AND India AND women; Title, abstract, keywords: chronic low back pain. lower back pain AND India AND women; Title, abstract, keywords: lower back pain AND women. backache AND India AND women. lumbar pain AND India AND women; lumbar pain AND women. pelvic pain AND India AND women. lumbago AND India AND women; lumbago. pelvic girdle pain AND India AND women. lumbopelvic pain AND India AND women.
PEDro Abstract title ''women india'' problem ''pain'' body part ''lumbar spine sacroiliac joint pelvis'' subdiscipline ''pain'' Abstract title ''women india'' problem ''pain'' body part ''lumbar spine sacroiliac joint pelvis'' subdiscipline ''ergonomics and occupational health''. Abstract title ''women india'' problem ''pain'' body part ''lumbar spine sacroiliac joint pelvis'' subdiscipline ''continence and women health''.
Cochrane library chronic pelvic pain in Title Abstract Keyword AND "India" in Keyword AND women in Keyword lower back pain in Title Abstract Keyword AND "India" in Keyword AND women in Keyword lumbar pain in Title Abstract Keyword AND "India" in Keyword AND women in Keyword lumbopelvic pain in Keyword AND "India" in Keyword AND women in Keyword.

Characteristics of LPP

Selection criteria of studies: Source [16]

Participants: Studies on Indian women with musculoskeletal pain in the lumbopelvic region irrespective of the chronicity of pain.

Studies selected: Studies addressing Indian women with LPP as the primary problem.

Studies excluded: Studies associating LPP with pregnant population; non-musculoskeletal origin; allopathy and complementary medicine; reviews and case studies.

Study design: All studies available that address Indian women, including peer and non-peer-reviewed gray literature and accepted manuscripts.

Outcome Measures

Lumbopelvic pain whether in the form of lumbar pain, pelvic pain, or a combination of the two.

Data Extraction

The recommendation of Chapter 7 of the Cochrane handbook [17] was followed for data extraction. A template was designed and pre-tested to obtain data relevant to this review. Disputes were resolved by authors through discussions. The following data were acquired: author details and publication year, title, study objective, research design, sample size, and characteristics, LPP description, LPP parameters assessed, outcome measure, and result.

Assessment of Risk of Bias

Qualities of non-experimental and experimental research articles were assessed through the Critical Appraisal Skills Programme (CASP) checklist and Cochrane risk of bias criteria for Effective Practice and Organization of Care reviews, respectively. Judgment for domains in CASP was assigned as Yes, Can’t tell, and No, where scores of 8-10, 5-7, and ≤4 meant high, moderate, and low-quality studies, respectively. Judgment of domains for Cochrane risk of bias criteria for effective practice and organization of care reviews were: high-risk studies, low-risk studies, and unclear (?), i.e. not specified in the article.

Data Synthesis

Data synthesis was narrative, as huge variations were observed between studies and were not appropriate for meta-analysis.

Results

**Figure 1:**
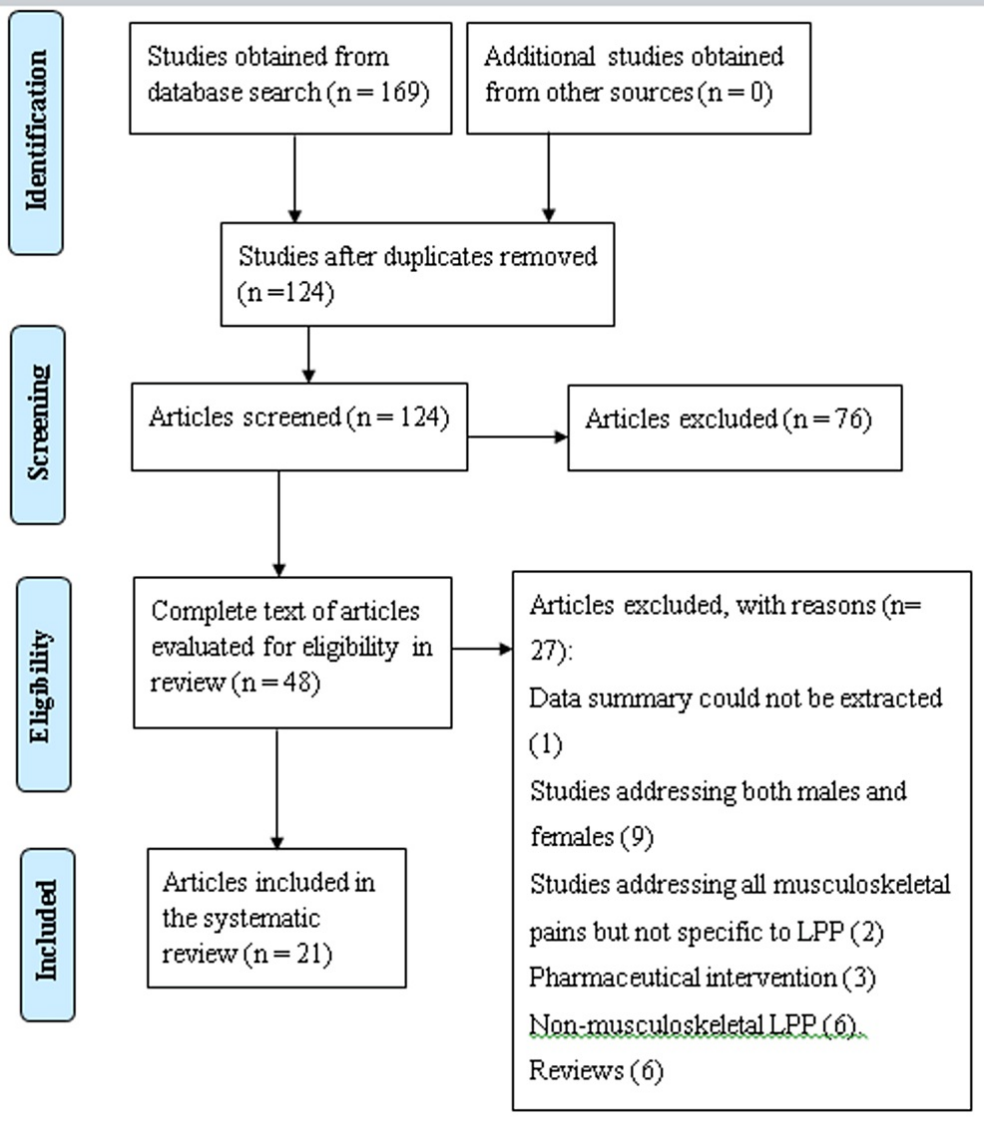
PRISMA flowchart PRISMA: Preferred Reporting Items for Systematic Reviews and Meta-Analyses

Overall, 169 LPP studies were retrieved from many databases (PubMed: 35; Pedro: 1, Science Direct: 124, Web of Science: 2, Google Scholar: 7). After removing the duplicate research articles, 124 study titles, as well as abstracts, were then screened. Full-text eligibility was assessed for 48 studies, which resulted in 21 articles (14 non-experimental and 7 experimental) for the review (Figure 1).

Risk of Bias

Overall, the quality of non-experimental (Table 2) and experimental (Table 3) studies was low.

**Table 2:** Critical appraisal skills program

Study	Clear statement of aim	Methodology appropriate	Research design appropriate	Recruitment strategy appropriate	Data addressed the issue	Researcher-participants relationship adequate	Ethical issues considered	Data analysis rigorous	Clear statement of findings	Research valuable	Study quality
Sushil et al, (2022) [18]	yes	yes	yes	yes	yes	yes	yes	yes	yes	yes	High
Nandi & Bhadra, (2018) [19]	yes	no	no	yes	yes	can’t tell	can’t tell	can’t tell	can’t tell	yes	Low
Ahdhi et al, (2016) [20]	yes	yes	yes	yes	can’t tell	can’t tell	yes	can’t tell	can’t tell	yes	Moderate
Gupta & Nandini, (2015) [21]	yes	yes	no	no	yes	can’t tell	yes	no	no	yes	Low
Das, (2015) [22]	yes	yes	yes	yes	yes	yes	yes	yes	yes	yes	High
Mitra, (2017) [23]	yes	yes	can’t tell	can’t tell	yes	can’t tell	can’t tell	no	can’t tell	yes	Low
Emmanuel & Ezhilarasu, (2016) [24]	yes	yes	yes	can’t tell	yes	can’t tell	yes	no	no	no	Low
Sachdeva et al, (2016) [25]	yes	yes	no	no	no	no	no	can’t tell	can’t tell	yes	Low
Shameela, (2015) [26]	yes	yes	yes	can’t tell	Yes	can’t tell	yes	can’t tell	can’t tell	yes	Moderate
Gangopadhyay et al, (2014) [27]	yes	yes	yes	can’t tell	yes	no	can’t tell	can’t tell	no	can’t tell	Low
Koley & Sandhu, (2009) [28]	yes	yes	yes	yes	yes	yes	Yes	yes	can’t tell	can’t tell	High
Shah et al, (2016) [29]	yes	yes	yes	no	yes	can’t tell	can’t tell	can’t tell	can’t tell	can’t tell	Low
Varte et al, (2013) [30]	yes	can’t tell	can’t tell	can’t tell	yes	no	can’t tell	can’t tell	yes	yes	Low
Koshy et al, (2020) [31]	yes	yes	yes	no	no	no	can’t tell	can’t tell	can’t tell	yes	Low

**Table 3:** Cochrane risk of bias criteria for Effective Practice and Organization of Care reviews

Study design	Random Sequence Generation Method	Intervention Allocation Concealment	Similarity of Baseline Outcome Assessment	Similarity of Baseline Characteristics	Participants and Personnel Blinding	Blinding of Outcome Assessors	Outcome Data incomplete?	Contamination?	Selective Reporting Bias	Other Sources of Bias. Analysis by Group Allocation	
											Quasi-experimental (Kale Shivaji, 2019)	?	High-risk	High-risk	?	?	?	?	High-risk	?	?	
Quasi-experimental (P., 2016)	?	High-risk	High-risk	?	?	?	?	High-risk	?	?	
Comparative (Dahiya et al, 2017)	?	?	High-risk	?	?	?	?	High-risk	?	?	
Randomized controlled single-blind design (Patil et al., 2018)	Low-risk	Low-risk	Low-risk	Low-risk	High-risk	Low-risk	Low-risk	Low-risk	Low-risk	Low-risk	
Comparative (Agarwal et al, 1996).	?	Low-risk	Low-risk	Low-risk	?	?	Low-risk	Low-risk	?	Low-risk	
Experimental (Sharma et al, 2017)	?	Low-risk	Low-risk	Low-risk	?	?	?	Low-risk	?	High-risk	
experimental (Krishnan, 2019)	?	?	High-risk	High-risk	?	?	?	?	?	?	

Prevalence

Overall, the point prevalence of LPP was 54.8% (95% C.I.: 40.9 - 68.7) % among Indian women, with a prevalence of 48.3% (95% C.I.: 31.9 - 64.7) % and 62% (95% C.I.: 31.6 - 93.6) % among women in the urban region and rural region, respectively.

Description of Pain Within the Lumbopelvic Region

The commonest term was ‘Low back pain’ (LBP). The majority of studies did not outline a well-defined pain location within the lumbopelvic region (Table 4).

**Table 4:** Prevalence and description of LPP among Indian women LPP: lumbopelvic pain

Sample	LPP terminology and description.	Prevalence of LPP
Urban women in Delhi (Sushil et al., 2022)	‘Lumbopelvic pain’, musculoskeletal pain in the lumbar spine and/or pelvic region.	
Urban working women (Varte et al., 2013)	'Low backache'; pain location not described.	57.7%.
Rural women in Pudducherry (Ahdhi et al., 2016)	'Low back pain’; pain location not described.	42%.
Rural housewives of Kanpur (Gupta & Nandini, 2015)	'Low back pain'; pain location not described.	83%.
Female brick workers in rural west Bengal (Das, 2015)	'Low back pain'; pain location not described.	70%
Non-working rural west Bengal (Mitra, 2017)	‘Low back pain’; pain location not described.	31% in the past 7 days and 40% had yearly LPP.
Nurses in a tertiary hospital in Vellore (Emmanuel & Ezhilarasu, 2016)	‘Low back pain'; pain location not described.	53.4 %
Women with pelvic inflammatory disease (Sachdeva et al., 2016)	‘Backache’; pain location not described.	86.4%
Female nurses (Shameela, 2015)	‘Low back pain'; pain location not described.	33%
35-55 years old women in Punjab (Koley & Sandhu, 2009)	'Low back pain’; pain location not described.	
50-60 years old postmenopausal s females (Shah et al., 2016)	'Low back pain'; pain location not described.	77%
Urban working women (Nandi & Bhadra, 2018)	'Back pain'; pain location not described.	25.30%
35-45 years old obese women (Koshy et al, 2020)	sacroiliac joint dysfunction	55%
Women from a village in Maharashtra (Kale Shivaji, 2019)	‘Low back pain'; pain location not described.	
Women from a village in Puducherry (P., 2016)	‘Low back pain'; pain location not described.	29 (72.5%)
Female teachers of primary school (Dahiya et al., 2017)	'Mechanical low back pain'; pain location not described.	
Nurses from a tertiary hospital in south India (Patil et al., 2018)	'Chronic low back’; pain site not described.	
Patients recruited from the gynecology department (Agarwal et al., 1996)	Chronic pelvic pain (CPP); "Intermittent or constant pain in the lower abdomen or pelvis of a woman, at least six months of duration’’	65.6% for 1-5 years, 18.8% for >5 years, and 15.6% for <1 year.
18-60 year-old women with CPP (Sharma et al., 2017)	'Chronic pelvic pain'; Clinically diagnosed.	
30 to 45 years old women with low back pain (Krishnan, 2019)	‘Lumbago'; Described as pain in the lumbar region or lower back.	

Characteristics of LPP

Pain intensity and disability associated with pain [19-23,26-29,32,33], were assessed through standardized questionnaires. Some authors prepared questionnaires to identify the duration, history, location, radiation to legs, aggravating or relieving factors, and any previous treatment/investigations.

Risk Factors

Multiple risk factors are listed below:

Biological: Increased age [20,23,24,30]; high body mass index (BMI) [23,24,30]. On the contrary, increased BMI was not a risk factor in the two articles [20,26].

Socioeconomic: Married women, marrying after 30 years of age, illiteracy, and low family income [20].

Ergonomic: Working in squatting, kneeling, and continuous sitting positions on an everyday basis [20-23,27,30].

Gynecological: Cesarean delivery, abortion, multiple deliveries [19,20], menopause [28], and pelvic inflammatory disease [25].

Biomechanical: Pronated foot posture [34], diastasis rectus abdominis [29].

Psychological factors: Dissatisfaction with the pay scale, monotony in the job [22,27]; Anxiety, depression, somatization, and hysteria [35,36].

Discussion

Overall, 21 studies were included in this review, where 14 were non-experimental and seven were experimental studies. According to the findings of this review, the prevalence of LPP is high among Indian women. Therefore, there is a need to conduct women-oriented research addressing LPP.

Most studies have not described the pain location within the lumbopelvic region [19,20,21-30,32-35]. As per the literature, about 60% of studies have not mentioned the site of lower back pain [4].

Risk Factors

It is suggested that LPP in women commonly coexists with gynecological, urologic, gastrointestinal, psychological, and musculoskeletal pathologies [36-40]. LPP risks specific to female reproductive function have been identified in this review [19,20,25,28]. Osteoporosis is the most common metabolic disorder that is known to accelerate degeneration in the lumbar spine discs. However, endocrinological pathology has been uninvestigated for LPP [41]. This clearly shows the severity of data deficiency on LPP in Indian women.

This review also observed ergonomic factors contributing to LPP, which were long hours of continuous sitting among urban working women [19,30]. While among rural women, LPP was common in those working as laborers, which are physically robust jobs with respect to strength and anthropometrics of women [22]. Further, certain jobs that are quite easy for men might be difficult for women’s anthropometrics, which needs women-specific ergonomic designs [12]. LPP was also reported by rural housewives who were not involved in any kind of physical activity except household chores [20,21]. In most Indian societies, women are the sole bearers of household chores, which involve sweeping the floors and washing utensils and clothes manually [20]. Bending of the trunk, squatting, and kneeling are associated with these household activities. Spending a substantial amount of time in such postures on an everyday basis can alter the biomechanical symmetry between innominate bones, reduce motor control of adjacent muscles, and/or cause trauma to lumbopelvic structures, which eventually leads to LPP [42,43]. Hence, there is a dire need to sensitize the deleterious effect of such habitual postures adopted by these women.

With a huge emphasis on the psychological aspect of LPP in recent literature [44], only work-related psychological risks were observed in the present review. It is noteworthy that these psychological risks were majorly reported by women working in physically exhausting jobs like chicken embroidery and laborers [22,27]. Occupations with extensive physical demands are known to affect the psychosocial life of LPP-afflicted individuals [45].

For the anthropometrics, high BMI was noted as the risk for LPP in this review [19,23,24,30], consistent with the literature [46,47]. Contrarily, two studies in this review showed no association between BMI and LPP [20,26]. However, there were only a few obese women (n=17) in one study [20], and the mean BMI was 20.8 in the other study [26]. The BMI-related data in this review is not sufficient to support its association with LPP.

An attempt was made to reduce the possibility of publication bias by conducting a comprehensive search for relevant articles. Moreover, the risk of bias was assessed for both experimental and non-experimental studies through appropriate tools.

Limitations

Only articles in the English language were included, which could have potentially missed important information about LPP in non-English language sources.

## Conclusions

The prevalence of LPP is high in Indian women. There is a severe deficiency in data on LPP, demonstrating an alarming need for research studies on the musculoskeletal as well as biopsychosocial consequences of LPP in Indian women. Further, ergonomic advice for women with LPP should be planned considering the needs and demands of their respective occupations as well as domestic chores since working women might spend a substantial amount of time at their workplace, but they too have a responsibility of household chores. During the risk of bias assessment, it was found that the overall quality of most of the studies was poor irrespective of the study designs. Though it was not the objective of this systematic review, it is a distinct finding, as it indicates a lacuna in data availability for LPP among women.

## References

[1] Schneider S, Randoll D, Buchner M (2006). Why do women have back pain more than men? A representative prevalence study in the federal republic of Germany. Clin J Pain.

[2] Balagué F, Mannion AF, Pellisé F, Cedraschi C (2012). Non-specific low back pain. Lancet.

[3] Bergström C, Persson M, Nergård KA, Mogren I (2017). Prevalence and predictors of persistent pelvic girdle pain 12 years postpartum. BMC Musculoskelet Disord.

[4] Hoy D, Bain C, Williams G (2012). A systematic review of the global prevalence of low back pain. Arthritis Rheum.

[5] Haldeman S, Nordin M, Outerbridge G (2015). Creating a sustainable model of spine care in underserved communities: the World Spine Care (WSC) charity. Spine J.

[6] Deshmukh SA, Kalkonde YV, Deshmukh MD, Bang AA, Bang AT (2014). Healthcare seeking behavior for back and joint pain in rural Gadchiroli, India: a population-based cross-sectional study. Indian J Community Med.

[7] Patel P, Das M, Das U (2018). The perceptions, health-seeking behaviours and access of Scheduled Caste women to maternal health services in Bihar, India. Reprod Health Matters.

[8] Hoy D, Brooks P, Blyth F, Buchbinder R (2010). The epidemiology of low back pain. Best Pract Res Clin Rheumatol.

[9] Meana M, Cho R, DesMeules M (2004). Chronic pain: the extra burden on Canadian women. BMC Womens Health.

[10] Banerjee A, Deaton A, Duflo E (2004). Wealth, health, and health services in rural Rajasthan. Am Econ Rev.

[11] Jones T, Kumar S (2001). Physical ergonomics in low-back pain prevention. J Occup Rehabil.

[12] Singh SP, Singh S, Singh P (2012). Ergonomics in developing hand operated maize dehusker-sheller for farm women. Appl Ergon.

[13] Driessen MT, Proper KI, van Tulder MW, Anema JR, Bongers PM, van der Beek AJ (2010). The effectiveness of physical and organisational ergonomic interventions on low back pain and neck pain: a systematic review. Occup Environ Med.

[14] Bernardes JM, Wanderck C, Moro AR (2012). Participatory ergonomic intervention for prevention of low back pain: assembly line redesign case. Work.

[15] Lumley MA, Cohen JL, Borszcz GS (2011). Pain and emotion: a biopsychosocial review of recent research. J Clin Psychol.

[16] Methley AM, Campbell S, Chew-Graham C, McNally R, Cheraghi-Sohi S (2014). PICO, PICOS and SPIDER: a comparison study of specificity and sensitivity in three search tools for qualitative systematic reviews. BMC Health Serv Res.

[17] Higgins JP, Green S. (2008). Cochrane Handbook for Systematic Reviews of Interventions: Version 5.1.0.

[18] Sushil P, Chawla JK, Kumar P, Duggal T (2022). Exploring Indian women’s perception and care seeking behavior towards lumbopelvic pain: a qualitative study. J Hum Behav Soc Environ.

[19] Nandi N, Bhadra B (2018). Low back ache in working women of reproductive age group in an urban area. New Indian J Obgyn.

[20] Ahdhi G, Subramanian R, Saya G, Yamuna TV (2016). Prevalence of low back pain and its relation to quality of life and disability among women in rural area of Puducherry, India. Indian J Pain.

[21] Gupta G, Nandini N (2015). Prevalence of low back pain in non working rural housewives of Kanpur, India. Int J Occup Med Environ Health.

[22] Das B (2015). An evaluation of low back pain among female brick field workers of West Bengal, India. Environ Health Prev Med.

[23] Mitra K (2017). Prevalence of low back pain and disability among the non-working adult women in a rural community of Purba Barddhaman, West Bengal. J Med Sci Clin Res.

[24] Emmanuel NM, Ezhilarasu P (2016). Low back pain among nurses in a tertiary hospital, South India. J Osteoporos Phys Act.

[25] Sachdeva P, Dahiya A, Singh R (2016). Incidence of pelvic inflammatory disease in backache in females. Int J Reprod Contracept Obstet Gynecol.

[26] Shameela TV (2015). Correlation of low back pain with body mass index, functional reach test among female nursing professionals. Int J Physiother.

[27] Gangopadhyay S, Chakrabarty S, Sarkar K, Dev S, Das T (2014). Evaluation of low back pain among female chikan embroidery workers of West Bengal. J Ind Eng Manag Innov.

[28] Koley S, Sandhu NK (2009). An association of body composition components with the menopausal status of patients with low back pain in Tarn Taran, Punjab, India. J Life Sci.

[29] Shah SK, Honkalas P, Kumar A (2016). Correlation of low back pain and diastasis rectus abdominis in post menopausal women between the age group of 50-60 years. Indian J Physiother Occup Ther - An Int J.

[30] Varte LR, Rawat S, Singh I, Majumdar D (2012). Duration of use of computer as risk factor for developing back pain among Indian office going women. Asian J Medi Cal Sci.

[31] Koshy MM, Patil P (2020). Prevalence of sacroiliac joint dysfunction in middle aged obese women. Indian J Public Heal Res Dev.

[32] Shivaji KK (2019). Effectiveness of yoga therapy on low back pain among women. Public Heal Open Access.

[33] Sumathy P (2016). Effectiveness of exercises on low back pain among middle aged women at Puducherry. J Health Allied Sci NU.

[34] Dahiya J, Rai R, Chugh P, Chopra C (2017). Role of foot posture in female school teachers with and without low back pain. Indian J Physiother Occup.

[35] Patil NJ, Nagaratna R, Tekur P, Manohar PV, Bhargav H, Patil D (2018). A randomized trial comparing effect of yoga and exercises on quality of life in among nursing population with chronic low back pain. Int J Yoga.

[36] Agarwal P, Khastgir U, Bhatia MS, Bohra N, Malik SC (1996). Psychological profile of females with chronic pelvic pain. Indian J Psychiatry.

[37] Sharma N, Rekha K, Srinivasan JK (2017). Efficacy of transcutaneous electrical nerve stimulation in the treatment of chronic pelvic pain. J Midlife Health.

[38] Krishnan GG (2019). Effect of yogic practices on elasticity among lumbago women. Int J Yogic Hum Mov Sports Sci.

[39] Montenegro ML, Gomide LB, Mateus-Vasconcelos EL, Rosa-e-Silva JC, Candido-dos-Reis FJ, Nogueira AA, Poli-Neto OB (2009). Abdominal myofascial pain syndrome must be considered in the differential diagnosis of chronic pelvic pain. Eur J Obstet Gynecol Reprod Biol.

[40] Sedighimehr N, Manshadi FD, Shokouhi N, Baghban AA (2018). Pelvic musculoskeletal dysfunctions in women with and without chronic pelvic pain. J Bodyw Mov Ther.

[41] Wang YX (2015). Postmenopausal Chinese women show accelerated lumbar disc degeneration compared with Chinese men. J Orthop Translat.

[42] Waddell G, Burton AK (2001). Occupational health guidelines for the management of low back pain at work: evidence review. Occup Med (Lond).

[43] Booth J, Morris S (2019). The sacroiliac joint - victim or culprit. Best Pract Res Clin Rheumatol.

[44] Pincus T, Kent P, Bronfort G, Loisel P, Pransky G, Hartvigsen J (2013). Twenty-five years with the biopsychosocial model of low back pain-is it time to celebrate? A report from the twelfth international forum for primary care research on low back pain. Spine (Phila Pa 1976).

[45] Vandergrift JL, Gold JE, Hanlon A, Punnett L (2012). Physical and psychosocial ergonomic risk factors for low back pain in automobile manufacturing workers. Occup Environ Med.

[46] Vismara L, Menegoni F, Zaina F, Galli M, Negrini S, Capodaglio P (2010). Effect of obesity and low back pain on spinal mobility: a cross sectional study in women. J Neuroeng Rehabil.

[47] Goyal L, Ajmera K (2022). Osteoporosis: a step-by-step case-based study. Cureus.

